# Chinese Marine Materia Medica Resources: Status and Potential

**DOI:** 10.3390/md14030046

**Published:** 2016-03-03

**Authors:** Xiu-Mei Fu, Meng-Qi Zhang, Chang-Lun Shao, Guo-Qiang Li, Hong Bai, Gui-Lin Dai, Qian-Wen Chen, Wei Kong, Xian-Jun Fu, Chang-Yun Wang

**Affiliations:** 1 xiumei@ouc.edu.cnbjb908@ouc.edu.cnliguoqiang@ouc.edu.cnbaihong78@163.com; 2 daiguilin@ouc.edu.cnchenqianwen1111@163.com15266209316@163.com; 3; 4 xianxiu@hotmail.com

**Keywords:** Chinese marine materia medica, marine medicinal bioresources, investigation, evaluation, drug development

## Abstract

Chinese marine materia medica (CMMM) is a vital part of traditional Chinese medicine (TCM). Compared with terrestrial TCM, CMMM, derived from specific marine habitats, possesses peculiar chemical components with unique structures reflecting as potent pharmacological activities, distinct drug properties and functions. Nowadays, CMMM appears to be especially effective in treating such difficult diseases as cancers, diabetes, cardio-cerebrovascular diseases, immunodeficiency diseases and senile dementia, and therefore has become an important medicinal resource for the research and development of new drugs. In recent years, such development has attracted wide attention in the field of medicine. In this study, the CMMM resources in China were systematically investigated and evaluated. It was found that the historic experiences of Chinese people using CMMM have continuously accumulated over a period of more than 3600 years, and that the achievements of the research on modern CMMM are especially outstanding. By June 2015, 725 kinds of CMMMs from Chinese coastal sea areas have been identified and recorded, covering 1552 organisms and minerals. More than 3100 traditional prescriptions containing CMMMs have been imparted and inherited. However, the number of CMMMs is less than the 8188 terrestrial TCMs, from more than 12,100 medicinal terrestrial plants, animals and minerals. In the future, the research and development of CMMM should focus on the channel entries (TCM drug properties), compatibility, effective ingredients, acting mechanisms, drug metabolism and quality standard. This study reveals the high potential of CMMM development.

## 1. Introduction

Traditional Chinese medicine (TCM), with its unique theories of Chinese medication and medical practices, plays an essential role in the entire history of world medicine, and has made notable contributions to the health of Chinese nationals and people of the world for thousands of years. As an important part of TCM, Chinese marine materia medica (CMMM), based upon oceanic medical materials, plays a vital role in preventing and treating diseases [1,2,3,4]. Marine *materia medica* (MMM) derived from marine environments possessing distinct drug properties and functions appears to be especially effective when treating difficult diseases, such as cancers, diabetes, cardio-cerebrovascular diseases, immunodeficiency diseases and senile dementia [5,6]. Currently, it has become an important medicinal resource for the development of new drugs used for preventing and treating difficult diseases [7]. In recent years, the research and development for CMMM is attracting extensive attention in the field of medicine. It has also become a major strategic demand for innovative drug development in China [8]. As China boasts rich marine bioresources and unique traditional Chinese medicine theories, CMMM may become a feature in research and development of marine drugs in China. For this reason, research and development of modern CMMM based on the TCM theories are very important and practically significant. In this study, we conducted a systemic investigation and evaluation of the CMMM resources in China to ascertain the status of the CMMM resources in China and provide resources and databases for future research and development of CMMM.

## 2. Results and Discussion

With institutional review board (IRB) approval and informed consent, CMMM categories were investigated mainly in the TCM national markets of China, including Anguo TCM Market in Hebei, Bozhou TCM Market in Anhui, Qingping TCM Market in Guangdong, and Yulin TCM Market in Guangxi. The investigation was also conducted in coastal drug markets and marine product markets. Diverse ways and methods [9,10,11] were applied for the investigation, such as studying the drug markets, questionnaire surveys, interviewing pharmacists, inspecting medicinal material factories, literature survey (more than 500 pieces of ancient medical literature and over 20,000 pieces of modern literature), and network information searching (10 databases).

### 2.1. Backtracking the Progress of CMMM

**Figure 1:**
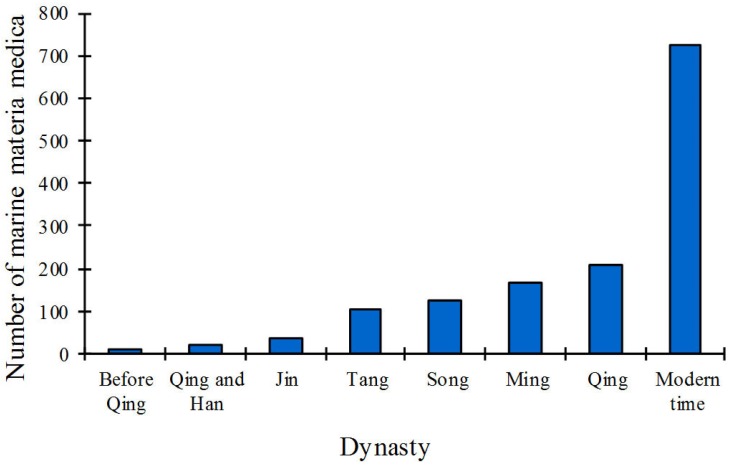
Developmental trend of Chinese marine materia medica (CMMM) in different dynasties of China.

By literature retrieval and study, we found that the history of using marine organisms as drugs in China could be traced back to the Shang period, about 3600 years ago. Ancient coastal residents first started investigating the edibility and medical use of marine organisms, marking a starting point for the clinical practice of marine drugs. By knowledge accumulation through long-term practices, Chinese ancestors learned to directly use some marine organisms and minerals as drugs. These medicinal experiences gradually accumulated in the form of classical writings during the past Chinese dynasties, especially in the form of medical books (Figure 1) [12].

According to *ShanHaiChing* [13], marine organisms were recorded in Xia and Shang dynasties (approximately1600 B.C.), concerning eight kinds of CMMMs, such as *Takifugu*, *Heterodontus*, and *Prognichthys et* *Cheilopogon* *et Exocoetus*. By Qin and Han dynasties (about 1800 years ago), more knowledge of marine medicines was acquired. Especially, *Shennong Bencaojing* (*Shennong’s Classic of Materia Medica*) [14]—an ancient book of Chinese herbology—mentioned 13 kinds of CMMMs, including *Sargassum*, *Chelonia* *Testudinis*, *Sepiae* *Endoconcha*, *Meretricis* *et Cyclinae* *Concha*, and *Eriocheir et Gaetice*. Thanks to the herbological development in Tang and Song dynasties, CMMM had achieved a remarkable progress in the Ming and Qing dynasties. *Tang Ben Cao* (*Tang* *Materia Medica*) [15], an imperial publication of the Tang dynasty (618–907), recorded 25 additional CMMMs, such as *Corallium*, *Cyrtiospirif*, *Notorynchi et Musteli* *Cortex*, *Mauritiae* *et Cypraeae* *Concha*, *Turbinis* *Operculum*, *Mactra et* *Lutraria*, *Silvetia*, and *Porphyrae* *Thallus*. *Compendium of Materia Medica* [16], a masterpiece compiled by the prominent herbalist Shizhen Li in the Ming dynasty (1368–1644) recorded 151 kinds of CMMMs, including such new varieties as *Macreophtalmus*, *Tylorrhynchus et* *Nectoneanthes* *et Neanthes*, *Gelidium*, and *Gracilaria*. *Supplement to Compendium of Materia Medica* [17] compiled by Xuemin Zhao in the Qing dynasty gave an account of 33 additional kinds of CMMMs, such as *Caloglossa* *Leprieurii*, *Eucheuma* *Denticulate* *Kappaphycus*, *Notarchi et* *Aplysiae* *Ovum* *Saccus*, *Trichiurus* *et Eupleurogrammus et* *Lepturacanthus*, and *Syngnathus*. In addition to the CMMM, the ancient books above also recorded thousands of formulas (prescriptions and dietary therapy formulas) with the CMMMs as principal drugs. During the latter half of the 20th century, new CMMMs have been consecutively discovered along with the rapid development of TCM and modern marine drugs. In recent decades, it was found that there are a large number of marine drug formulas with CMMMs commonly applied in Chinese coastal areas, including proved formulas, secret prescriptions and folk formulas. These prescriptions and formulas have been dug out by long time investigation and practice, and their clinical effects have been verified in modern clinical use by traditional Chinese medicine physicians. As a result, these proved and effective recipes have been recorded in modern medical books, for instance, *Chinese Materia Medica* [18], and *Chinese Marine Materia Medica* [1]. The latest *Chinese Marine Materia Medica* [1], for example, comprises 613 CMMMs and 3100 formulas with the compatibility of CMMMs as principal drugs, providing a unique resource base for the research and development of modern CMMM drugs.

### 2.2. Investigation of CMMM Resources in China

The CMMM resources were investigated by field inspection and literature survey. The field investigation focused on four main areas, including the tropical coral reef ecosystem, the tropical and subtropical mangrove ecosystem, the estuary and intertidal zone ecosystem, and the aquatic ecosystem surrounding islands. The tropical coral reef ecosystem with extremely prolific biodiversity has become a vital biological source of marine natural products and modern marine drugs. The tropical and subtropical mangrove ecosystem features abundant of medicinal mangrove forests as well as epiorganisms or symbionts. The estuary and intertidal zone ecosystem, a coastal zone connecting with land and fresh waters, is the most convenient place for people to obtain and utilize medicinal marine organisms, while the aquatic ecosystem surrounding islands is less influenced by human activities, and thus the biocommunity maintains a native ecological state. Our investigation and statistical analysis indicated that CMMM resources are distributed within all Chinese seas, including the Bohai Sea, the Yellow Sea, the East China Sea, and the South China Sea, covering coastal areas and wetlands in 58 counties under the jurisdiction of 14 provinces, cities and special administrative regions (*i.e.*, Liaoning, Hebei, Tianjin, Shandong, Jiangsu, Shanghai, Zhejiang, Fujian, Taiwan, Guangdong, Hong Kong, Macao, Hainan, and Guangxi). Some marine medicinal species only exist in China, and some of them are labeled as world rare species. By June 2015, a total of 725 CMMMs have been recorded and studied in terms of modern pharmacology and chemistry, involving 1552 species of medicinal organisms and minerals (Table 1). The category of medicinal marine organisms falls into 17 biological taxa, consisting of one phylum of cyanobacteria; four phyla of algae; two phyla of salt marsh vascular plants, mangroves and seashore associated embryophytes; and 10 phyla of animals. For the medicinal cyanobacteria, 13 species are included. There are four phyla of algae including red algae, brown algae, diatom and green algae and two phyla of plants from coastal wetlands including Pteridophyta and Angiospermae. China has 171 species of medicinal seaweeds, accounting for 11.1% of the total medicinal marine organisms, including 94 species of red seaweed and 45 species of brown seaweed. There are 80 species of medicinal wetland plants, and 23 of them are medicinal mangrove plants growing in mangrove ecosystem at land–sea junctures [19]. Among the 10 phyla of medicinal marine animals, there are seven phyla of medicinal marine invertebrates including Porifera, Coelenterata, Annelida, Sipuncula, Mollusca, Arthropoda, and Echinodermata, as well as Urochordata, Cephalochordata and Chordata. Medicinal invertebrates account for 45.1% of the total medicinal marine organisms, with Mollusca being ranked first, including up to 428 species. In addition, there are 574 species of medicinal Chordata, up to 37.4% of the total medicinal marine organisms. It should be noted that 16 medicinal endemic species have been identified in Chinese seas (Table 2), and 76 poisonous species in medicinal species have been recorded. Especially, there are 33 gorgonians and soft corals that belong to the hot species for the studies of modern marine drugs showing a great prospect in medicinal applications [20]. These medicinal marine organisms are distributed in the vast area of Chinese seas, showing an incremental trend of the species numbers from the north to the south of Chinese coastal areas. The resources of some marine medicinal organisms are prolific, ensuring sufficient medicinal supplies for drug development, such as *Laminariae* *Thallus*, *Porphyrae Thallus*, *Haliotidis Musculus*, *Haliotidis Concha*, and *Margarita*. The endangered species, such as *Syngnathus*, *Hippocampus*, and *Takifugu*, come to a total number of 92 species. Most of the traditional Chinese medicine materials have been produced using artificial aquaculture as well as from wild collection. For example, the original species of medicinal *Hippocampus* (seahorses), *Laminariae*, *Ostreae*, and *Margarita*, have been artificially bred in large scale for drug use [21].

### 2.3. Research and Application of CMMM

Commodity market investigation indicated that among the 725 kinds of CMMMs, 50 kinds are in common medicinal use, including 34 marine animal drugs, 13 marine plant drugs, and three marine mineral drugs (Table 3), all of which are derived from oceanic environments and widely used by the residents in the coastal areas of China. Most of the CMMMs are traditional medicines with unique functions for special use [34,35,36,37,38,39,40], such as *Laminariae Thallus*, *Porphyrae Thallus*, *Digenea Thallus*, *Sepiae Endoconcha*, *Hippocampus*, *Syngnathus*, *Placunae et Enigmoniae et Anomiae Musculus*, *Haliotidis Concha*, *Arcae Concha*, *Meretricis et Cyclinae Concha*, *Apostichopus et Stichopus et Thelenota*, and *Anthocidaris et Hemicentrotus et Strongylocentrotus Concha*.

**Figure 2:**
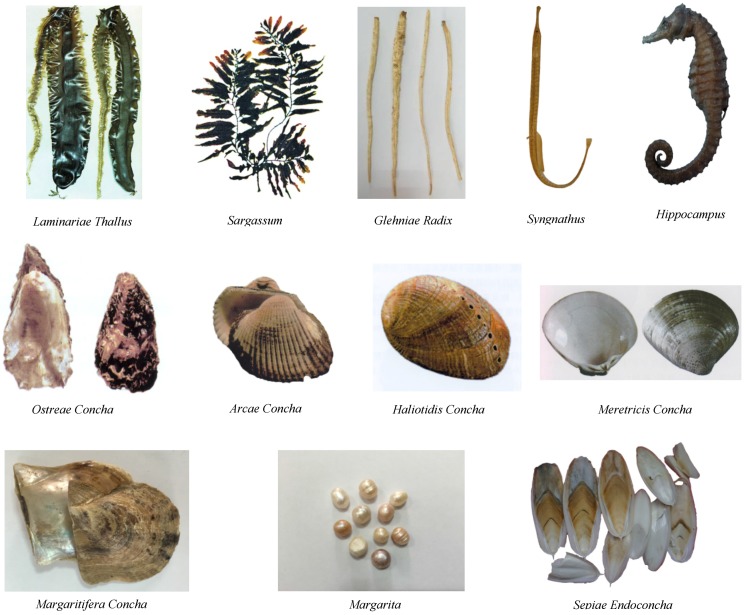
The CMMMs recorded in the Pharmacopoeia of People's Republic of China [1,41].

**Figure 3:**
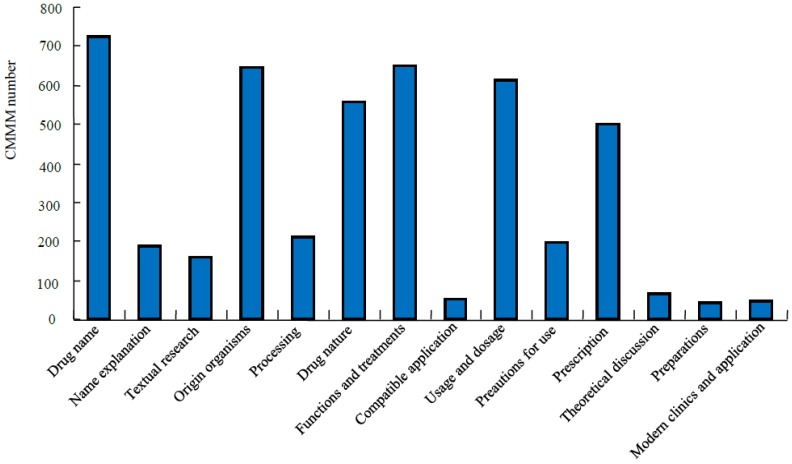
Records of CMMMs in the past Chinese dynasties and the status of its modern research.

**Figure 4:**
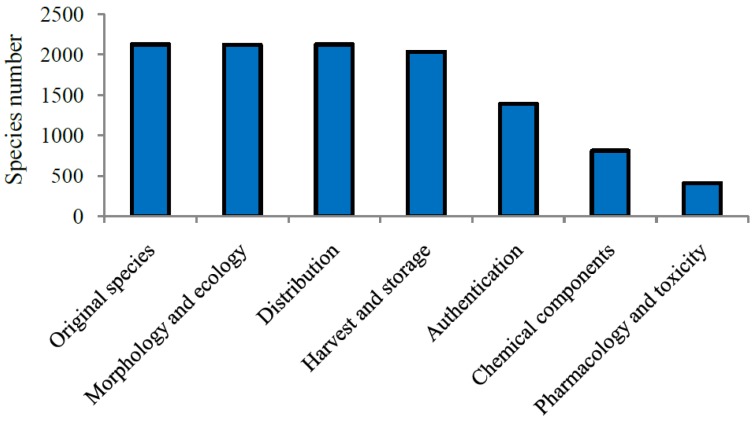
The status of research on the original organisms of CMMMs.

Specifically, 12 CMMMs have been recorded in the *Chinese Pharmacopoeia* (2015) [41], which comprise nine animal MMMs (*Arcae Concha*, *Haliotidis Concha*, *Ostreae Concha*, *Margaritifera Concha*, *Meretricis Concha*, *Hippocampus*, *Syngnathus*, *Sepiae Endoconcha*, and *Margarita*) and three botanic MMMs (*Sargassum*, *Laminariae Thallus*, and *Glehniae Radix*) (Figure 2). The Medicament portions of CMMMs often contain the whole alga, the shell, meat, and bone of animals with different drug properties to produce unique efficacies. Compared to the typical “terrestrial TCM”, CMMM (marine TCM) is characterized with peculiar drug properties and channel entries. Most frequently-used CMMM drugs are generally cold but seldom warm in nature; and their flavors tend to be salty or sweet [42]. Studies have shown that salty-cold (such as *Sargassum* and *Laminariae Thallus*), salty-warm (*Hippocampus* and *Syngnathus*), sweet-neutral (*Apostichopus* *et* *Stichopus* *et* *Thelenota* and *Chlamydis* *et* *Mimachlamydis* *et* *Argopectinis Adduotor*) are representative in the natures and flavors of CMMMs, with salty flavor up to more than 90% of the total flavors. In terms of medicinal effects, the most representative efficacies of CMMMs include softening hard mass and removing stasis (*Sargassum*, *Laminariae Thallus*, *Ostreae Concha*, and *Meretricis Concha*), removing heat to cool blood, neutralizing poison and eliminating purpura (*Porphyrae Thallus*, *Gelidium*, and *Gracilaria*), invigorating kidney and strengthening essence (*Hippocampus*, *Syngnathus*, and *Callorhini* *et* *Phocae Nephros*), and calming liver and suppressing Yang (*Haliotidis Concha* and *Margaritifera Concha*) [1,43]. Therefore, the medicinal use of CMMM is wide, especially in the effective treatment of serious, difficult, and complex diseases, such as cancers, cardiovascular and cerebrovascular diseases and diabetes mellitus. With the development of life science and technologies, the contemporary research on CMMM is in gradual progress. Among the 725 CMMMs, the categories that have been studied for processing, compatible application, preparations and modern clinical observation are 209, 53, 40 and 45, respectively (Figure 3). Within the 1552 medicinal marine species, 736 and 381 have been investigated for chemical components and pharmacology and toxicity, respectively (Figure 4).

In recent decades, the efficacies of CMMMs have been verified continuously by modern clinic research. For example, *Laminariae* is a very common CMMM used in China. Its functions mainly focused on dissolving phlegm, relieving cough and asthma, softening hard mass and eliminating stagnation, and promoting diuresis and dredging stranguria. The Capsule of *Laminariae Thallus* powder was used for treating hypertensive patients of I–II period. The systolic pressure and diastolic pressure of the patients were effectively reduced when they took 12 g *Laminariae Thallus* powder every day. The Capsule of *Laminariae Thallus* powder was found to enhance the function of conventional antihypertensive drugs when co-administrated [37]. *Arcae Concha* is also a common CMMM possessing the efficiency of dissolving phlegm, breaking stagnate and relieving pain. It has been reported that the superfine powder of *Arcae Concha* was applied exteriorly for different levels of chilblain, and all 35 patients recovered after 2–6 times [38].

In most cases, the CMMMs are usually used with other TCMs by compatibility rule in traditional Chinese prescriptions. Ulcer Styptic Powder, a compound recipe consisting of *Sepiae Endoconcha* and *Bletillae Rhizoma* with a ratio of 2:1, was applied to treat 100 patients of hematemesis or hematochezia induced by duodenal ulcer. The patients took Ulcer Styptic Powder, 2–4 g or 4–6 g per time, and 3–4 times one day. The results indicated that 97 patients stopped bleeding or recovered from hematemesis or hematochezia [39]. *Rhei Radix* *et Rhizoma* and *Ostreae Concha* Decoction was prepared with 30 g calcine *Ostreae Concha*, 30 g fresh *Rhei Radix* *et Rhizoma*, and other TCM materials. This decoction was used to treat 38 uremia patients by high retention-enema of rectum with 200 mL one day. A 10-day treatment course was designed, and the treatment period was 1–3 courses depending on the patient situation. The results revealed that this prescription was effective for 33 cases, of which 17 cases exhibited excellent effects [40].

Specifically, the studies on the effective components of CMMMs have also been executed by modern technology in recent decades. From most CMMMs, the main chemical components have been identified and analyzed and their pharmacological activities have been tested and evaluated. A series of effective constituents have been extracted and developed as modern component drugs. The most famous example is the research and development of the *Laminariae*, a traditional CMMM from brown alga. Chemical component analysis indicated that *Laminariae* contains abundant laminarin including algin, fucoidan and laminaran, as well as other ingredients, such as mannitol, iodine and inorganic elements. *Laminariae* is a rich source of algin, mannitol and iodine. Several modern drugs have been developed from *Laminariae*. Particularly, based on the main component algin, Alginic Sodium Diester (PSS^®^) was developed into the market in 1985 as a marine polysaccharide sulfated drug with anti-hyperlipidemia function [44,45]. Up to date, Alginic Sodium Diester has been widely applied in clinic [46]. Mannitol Nicotinate is another marine drug with angiectasis and anti-hyperlipidemia effects developed based on mannitol extracted from *Laminariae* [47]. In addition, Longmu Zhuanggu Granules^®^, an OTC calcium replenisher against rachitis and osteomalacia, is also a widely used marine prescription with calcium extracted from *Ostreae Concha* [48].

Currently, 20 single CMMM preparations and 200 compound CMMM preparations have been developed into the market. Nevertheless, it should be noted that the majority of the 3100 ancient marine herbal formulas and proved formulas as recorded in *Chinese Marine Materia Medica* have not been developed by modern technology. It may be predicted that the potential resources of CMMMs that have not yet been identified or utilized will be discovered and studied in the coming decades. These resources will no doubt become a prolific source for development of CMMMs under the guidance of Chinese traditional medicine theory, as well as a strategy resource to exploit modern marine drugs by high technology [12,49].

## 3. Methods

### 3.1. Investigation of CMMMs

With IRB approval and informed consent, CMMMs were investigated mainly in the TCM national markets of China, including Anguo TCM Market in Hebei, Bozhou TCM Market in Anhui, Qingping TCM Market in Guangdong, and Yulin TCM Market in Guangxi. The investigation was also conducted in coastal drug markets and marine product markets. Diverse ways and methods [9,10,11] were applied for the investigation, including samples and information collection, questionnaire surveys, interviewing pharmacists, and inspecting medicinal material factories. The questionnaire surveys covered CMMM assortments, original organisms, production areas, resource status, medicament portions, processing methods, storage conditions, storage duration, quality grades, prices, usages, outputs, sales volumes, and retailers (see Supplementary Information). In the factories, the productions of CMMMs were inspected, including CMMM assortments, original organisms, habitats, resource status, processing methods, storage conditions, outputs, and quality grades.

The literature survey was conducted by searching ancient medical literature and modern databases. The ancient medical literature looked up covered 500 pieces of traditional Chinese Medicine text concerning CMMMs. Ten databases were used as the main origin of medicinal information, including Traditional Chinese Medical Database System, Scientific Database of China Plant Species, Scientific Database of China Animal Species, Medline, Marinlit, ScienceDirect, SpringerLink, Wiley Online Library, ACS Publication, and RSC Publication. The data and information were collected and extracted from over 20,000 pieces of modern literature, including CMMM assortments, original organisms, chemical components, pharmacological activities, toxicity, clinical application, production areas, resource status, medicament portions, processing methods, storage conditions, storage duration, quality grades, prices, usages, outputs, and sales volumes.

The investigation was focused on the common endemic CMMMs in China. The original data were collected, summarized and analyzed. The inaccurate or invalid data were removed, and the valid data were transformed to charts using Excel and SPSS. The CMMM status was assessed based on the above data and information.

### 3.2. Investigation of Original Organisms of CMMMs

The fieldwork and historical document survey were carried out to investigate the original organisms of CMMMs and the distribution of marine medical species in China. A specific field investigation on marine medicinal bioresources was conducted in the South China Sea, the East China Sea, the Yellow Sea, and the Bohai Sea. The main areas for field investigation were selected based on the features of the Chinese oceanic geographical environment, marine ecosystem and marine medicinal bioresources, including tropical coral reef ecosystem, tropical and subtropical mangrove ecosystem, estuary and intertidal zone ecosystem, and aquatic ecosystem surrounding islands. The tropical coral reef ecosystem areas covered the South China Sea and those coastal waters near Hainan, Guangdong and Guangxi provinces. Specifically, the medicinal marine organisms were collected in the sea areas around Hainan Island (near Sanya, Lingshui, Wanning, Wenchang, Qionghai, Lingao, and Danzhou), Leizhou Peninsula (near Xuwen), Naozhou Island, Weizhou Island, and Xisha Islands. The key investigation areas of tropical and subtropical mangrove ecosystem included Qiongshan in Hainan (Dongzhaigang National Mangrove Natural Reserve), Wenchang in Hainan (Qinglangang Mangrove Natural Reserve), Sanya in Hainan (Yalongwan Mangrove Natural Reserve, and Sanya Estuary Mangrove Natural Reserve), Danzhou in Hainan (Xinying Mangrove Natural Reserve), Lingao in Hainan (Xinying Mangrove Natural Reserve), Zhanjiang in Guangdong (Zhanjiang Mangrove Natural Reserve), Hepu in Guangxi (Shankou National Mangrove Natural Reserve), Leizhou Peninsula, Xiamen and Longhai in Fujian, and Wenzhou in Zhejiang. The key investigation areas in estuary and intertidal zone ecosystem included roundabout Hainan Island, coastal areas of Guangdong, Guangxi, Zhejiang, and Shandong, Bohai gulf, and estuaries of Zhujiang River, Yangtze River, and Yellow River. The key investigation areas of the aquatic ecosystem surrounding islands included the sea areas around Weizhou Island, Naozhou Island, Nanao Island, Nanji Island, Zhoushan Islands, Lingshan Island and Miaodao Islands. The collection methods for the marine organisms included trawling sampling (horizontal trawling, slant trawling, and vertical trawling), bottom characteristics sampling, and SCUBA diving sampling [50,51]. From March 2006 to June 2015, field investigation was conducted on 15 voyages, involving 400 sites in 170 cross-sections around the Chinese Seas. In total, 18,445 biological samples were collected from various marine habitats. All of the collected samples were identified for their biological species. The extracts of all the samples were evaluated for their bioactivities by bioassay approaches with pharmaceutical screening models. Up to 5767 species were identified, among which 1552 species were clarified as the medicinal species.

### 3.3. Identification of Original Species of CMMMs

Medicinal coral species were identified by Hui Huang and Xiubao Li from South China Sea Institute, Chinese Academy of Sciences, China; medicinal sponge species were identified by Nicole J. de Voogd from Netherlands Centre for Biodiversity Naturalis, The Netherlands; medicinal mangrove species were identified by Cairong Zhong from Dongzhaigang National Mangrove Natural Reserve, China; medicinal algae were identified by Shuben Qian from Ocean University of China, and Bangmei Xia from Institute of Oceanology, Chinese Academy of Sciences, China; medicinal coastal wetland plants were identified by Fengqin Zhou from Shandong Traditional Chinese Medicine University, China; and medicinal fish, shellfish, and other invertebrates were identified by Yunfei Wu, Xiaoqi Zeng, Shichun Sun and Zhenjiang Ye from Ocean University of China, and Daohai Chen from Zhanjiang Normal University.

### 3.4. Investigation of Folk Application of CMMMs

The folk interviews included personal interview, telephone interview, conversazione, and consulting to fishers and folk doctors. The folk recipes, nostrums and informal prescriptions using CMMMs were collected and uncovered from Chinese coastal areas, mainly in coastal villages and small towns. The folk investigation and interview routes were along the Chinese southeast coastlines passing through the residential communities, involving Liaoning, Hebei, Shandong, Zhejiang, Guangdong, Guangxi, and Hainan provinces.

## 4. Conclusions

In present study, CMMM resources in China were investigated by systematical evaluation and statistical analysis. It was found that there are totally 725 kinds of CMMMs, covering 1552 original medicinal marine plants, animals and minerals. More than 3100 prescriptions containing CMMMs have been used with compatibility. CMMM, possessing distinct drug properties and functions, has been proven to be especially effective for treating chronic, complex and difficult diseases. It has now become an important medicinal resource for the research and development of drugs. From a perspective of historical origin of CMMM research and application, we know that CMMM, which has become an integral part of TCM treasury, plays a unique role in the history of TCM development. It is imperative to comprehend its essence from modern views, interpret its mystery with modern science, and dig into its potential using modern technology. Many CMMMs have been proven their medical usage as recorded in ancient TCM literature and also turned out to be effective after long-term clinical practices. Nevertheless, compared with the terrestrial TCM, from an overall view, the level of CMMM development is relatively low. The knowledge of CMMM as a special TCM resource is insufficient, while the research and development of its modern application is still in the infancy. Therefore, in future research and development of CMMMs, the key approaches to study should be the nature of the drugs, channel entries, compatible applications, efficient substances, action mechanisms, drug metabolism and quality standards. The development of marine compound preparations and component drugs under the guidance of TCM theories using CMMMs and their formulas could be expected.

## Figures and Tables

**Table 1:** The status of marine *materia medica* resources in China.

Category	Phylum	Medicinal Resources	
		*Materia Medica* No.	Medicinal Species No.
Cyanobacteria	Cyanophyta	6	13
Algae	Rhodophyta	48	94
	Bacillariophyta	2	8
	Phaeophyta	23	45
	Chlorophyta	10	24
Salt marsh vascular plants, mangroves, and seashore associated embryophytes	Pteridophyta	1	1
	Angiospermae	114	79
Marine animals	Porifera	9	9
	Coelenterata	42	56
	Annelida	4	8
	Sipuncula	2	3
	Mollusca	111	428
	Arthropoda	33	106
	Echinodermata	19	83
	Urochordata	3	4
	Cephalochordata	1	1
	Chordata	285	574
Marine minerals	Minerals	12	16
In total	-	725	1552

**Table 2:** The endemic medicinal marine species in Chinese seas.

No.	Sorts	Species	Main Distribution Area
1	Bivalve	*Lithophaga curta* Lischke	The Chinese seas [22,23]
2	Crustacea	*Panulirus stimpsoni* Holthuis	The East China Sea and the northern part of the South China Sea [22,24]
3	Echinoderm	*Temnopleurus hardwickii* (Gray)	The Bohai Sea, the Yellow Sea, and the East China Sea [22,25]
4		*Temnotrema sculptum* (A. Agassiz)	Taiwan Strait [22,26]
5	Fish	*Psephurus gladius* (Martens)	Estuaries of the Yangtze River and Qiantangjiang River [22,27]
6		*Centrophorus niaukang* Teng	The eastern coastal area of Taiwan [22,28]
7		*Hydrolagus tsengi* (Fang *et* Wang)	Sea areas from Shandong to Zhejiang provinces [22,28]
8		*Acipenser sinensis* Gray	Estuaries of the rivers to the Yellow Sea, the East China Sea, and the South China Sea [22,27]
9		*Osteomugil affinis* (Günther)	The East China Sea and the South China Sea [22,29]
10	Algae	*Porphyra guangdongensis* Tseng *et* T. J. Chang	Sea area of Fujian and Guangdong provinces [22,30]
11		*Gracilaria rubra* Chang *et* Xia	Sea area of Hainan province [22,31]
12		*Laurencia jejuna* Tseng	Sea area of Hong Kong [22,32]
13		*Laurencia longicaulis* Tseng	Sea area of Hong Kong [22,32]
14		*Sargassum emarginatum* Tseng *et* Lu	Sea area of Xisha Islands [22,33]
15		*Sargassum phyllocystum* Tseng *et* Lu	Sea area of Xisha Islands [22,33]
16		*Turbibaria parvifolia* Tseng *et* Lu	Sea area of Xisha Islands [22,33]

**Table 3:** Common varieties of CMMMs and their original species.

No	*Materia Medica*	Herbal Nature	Functions and Treatments	Medicament Portions	Original Species	Main Distribution Areas
**Cyanobacteria**						
1	*Spirulina*	Sweet and salty in flavor, cool-natured	Nourishing and strengthening body, strengthening spleen and nourishing stomach, invigorating kidney, reducing blood lipids, reducing adverse reactions of radiotherapy and chemotherapy in cancer. Mainly treating digestive tract ulcer, iron-deficiency anemia, hyperlipidemia, diabetes, chronic liver disease, malnutrition, weakness after illness, adjuvant therapy for cancer.	Frond	*Spirulina platensis* (Notdstedt) Geitler	Widely distributed in warm sea areas; have been artificial cultured in industry in China.
**Algae**						
2	*Gelidium*	Sweet and salty in flavor, cold-natured	Clearing heat and detoxifying, Clearing lung and eliminating phlegm, dispersing blood stasis, lubricating intestines, eliminating piles and stopping bleeding, dispelling ascarid. Mainly treating lung-heat and expectoration, goiter and tumor, crewels, enteritis, diarrhea, chronic constipation, ascariasis, bronchitis, nephropyelitis, piles bleeding, tumor.	Frond	*Gelidium amansii* (Lamouroux) Lamouroux	The Bohai Sea, the Yellow Sea, and the East China Sea, China; Russia, Japan, and Korean Peninsula.
					*Gelidium crinale* (Turn.) Gaillon	The coastal area of China; the west coast of the Pacific Ocean from Japan to Vietnam.
					*Gelidiella acerosa* (Forssk.) Feldm. *et* Hamel	The sea areas of Hainan, Xisha Islands, and Taiwan, China; the west Pacific Ocean, and Indian Ocean.
3	*Eucheuma denticulate Kappaphycus*	Salty, mild-natured	Clearing heat and eliminating phlegm, softening hard mass and eliminating stagnation, relieving cough, eliminating hemorrhoids. Mainly treating phlegm-heat and cough, piles, goiter and tumor, crewels, trachitis, pneumonia.	Frond	*Eucheuma denticulatum* (N. L. Burman) Collins *et* Hervey	The sea areas of Xisha Islands, and Taiwan, China; Japan, Malaysia, and Indonesia.
					*Kappaphycus cottonii* (Weber-van Bosse) Doty	The coastal areas of Qionghai country in Hainan, Xisha Islands, and Lanyu Islands of Taiwan, China; the Ryukyu Islands; Philippines, Guam, and Tanzania.
					*Kappaphycus striatum* (Schmitz) Doty	The sea areas of Hainan, and Xisha Islands, China; Mozambique, Indonesia, and Malaysia.
4	*Gracilaria*	Sweet and salty in flavor, cold-natured	Softening hard mass and eliminating stagnation, dissipating phlegm, clearing heat and detoxifying, inducing diuresis, purgation. Mainly treating calor internus and subcutaneous nodule, qi stagnation due to goiter and tumor, difficult urination, urine sting, bronchitis, thyroid swelling, enteritis and dysentery, chronic constipation.	Frond	*Gracilaria asiatica* Zhang *et* Xia	The coastal area of China; Russia, Japan, and Korean Peninsula.
					*Gracilaria chouae* Zhang *et* Xia	The coastal areas of Fujian, and Hainan, China; Japan, and Korean Peninsula.
					*Gracilaria blodgettii* Harvey	The coastal areas of Fujian, Guangdong, Hainan, and Taiwan, China; Japan, America, Australia, and Brazil.
					*Gracilaria eucheumoides* Harvey	The coastal areas of Hainan, and Taiwan, China; Japan, Vietnam, Thailand, Philippines, and Indonesia.
					*Gracilaria lemaneiformis* (Bory) Weber-van Bosse	The coastal area of Shandong, China; Philippines, Tailand, Costa Rica, Columbia, Canada, and Britain.
5	*Laminariae Thallus*	Salty, cold-natured	Dissolving phlegm, relieving cough and asthma, softening hard mass and eliminating stagnation, promoting diuresis and dredging stranguria. Mainly treating qi stagnation due to goiter and tumor, crewels, dysphagia, cough, morbid leukorrhea, nocturnal emission and semen spillage, dropsical beriberi, malignant sore, goiter, lymphonodi cervicales swelling, splenohepatomegalia and ascites, stranguria, chronic bronchitis, testis swelling and pain, hypertension, arteriosclerosis, senile cataract.	Thallus	*Laminaria japonica* Aresch.	The coastal areas of Liaodong Peninsula and Shandong Peninsula; cultivated in Zhejiang, Fujian, and Guangdong, China; Russia, Japan, and Korean Peninsula.
					*Ecklonia kurome* Okam.	The coastal areas of Yushan Island of Zhejiang and Pingtan Island of Fujian, China; Japan, and Korean Peninsula.
					*Undaria pinnatifida* (Harv.) Sur.	The coastal areas of Liaoning (Lvda), Shandong (Yantai, Weihai, Rongcheng, and Qingdao), and Zhejiang (Shengsi islands); Japan, and Korean Peninsula.
6	*Macrocystis*	Salty, cold-natured	Clearing heat and eliminating phlegm, softening hard mass and eliminating stagnation, inducing diuresis to alleviate edema. Mainly treating goiter, lymphonodi cervicales swelling, trachitis, hypertension.	Frond	*Macrocystis pyrifera* (L.) C. Agardh	Aquicultured in the sea areas of Liaoning (Dalian), and Shandong (Changdao), China; the west coastal areas of North America and South America.
7	*Sargassum*	Salty, cold-natured	Dissolving phlegm, inducing diuresis, softening hard mass and eliminating stagnation, detumescence, discharging heat. Mainly treating goiter and tumor, crewels, intestine obstruction and accumulation, carbuncle and furunculosis, phlegm and fluid retention, edema, disuria and urine retention, micturition disorders, hypertension, hyperlipidemia, angina pectoris, skin disease, myoma of uterus, testis swelling and pain, iodine deficiency disease, tumor.	Frond	*Hizikia fusiforme* (Harv.) Okamura	The coastal area of China; Japan, and Korean Peninsula.
					*Sargassum confusum* C. Ag.	The Bohai Sea, the Yellow Sea, China; Russia, Japan, and Korean Peninsula.
8	*Enteromorpha*	Salty, cold-natured	Softening hard mass and eliminating stagnation, dissipating phlegm and removing qi stagnation, clearing heat and detoxifying, detumescence and hemostasis. Mainly treating goiter and tumor, crewels, abdominal distension, food retention, intestinal parasitosis, dysphoria, sore and furuncle, swelling, carbuncle on the back.	Frond	*Enteromorpha prolifera* (Muller) J. Agardh	The coastal area of China; Russia, and Japan.
					*Entermorpha clathrata* (Roth) Greville	The coastal areas of Zhejiang, Fujian, Guangdong, Hainan, and Taiwan; Russia, and Japan.
					*Entermorpha compressa* (L.) Grev.	The Bohai Sea, the Yellow Sea, and Taiwan, China; the west and east coastal areas of the Pacific Ocean, Indian Ocean, Red sea, and Mediterranean.
					*Entermorpha linza* (L.) J. Ag.	The coastal area of China; Russia, Japan, Korean Peninsula, and Vietnam.
					*Entermorpha flexuosa* (Wulf.) J.Ag.	The coastal areas of Guangdong, and Hainan, China; Japan to Malaya Peninsula, California coast of America to Ecuador, Polynesia, Oceania, and Atlantic Ocean.
					*Entermorpha intestinalis* (L.) Link	The coastal area of China, Russia, and Japan.
9	*Ulva*	Salty and sweet in flavor, cold-natured	Inducing diuresis to alleviate edema, softening hard mass and eliminating stagnation, clearing heat and detoxifying, dissipating phlegm, decompression.	Frond	*Ulva lactuca* Linnaeus	The coastal areas from Zhejiang to Hainan, China; Japan, and Vietnam.
					*Ulva pertusa* Kjellman	The Bohai Sea, and the Yellow Sea, China; Russia, Korean Peninsula, and Japan.
					*Ulva fasciata* Delile	The coastal area of Guangdong, China; The Ryukyu islands, Malaysia, the east coast of Pacific Ocean, and Oceania.
**Salt marsh vascular plants, mangroves and seashore associated embryophytes**						
10	*Suaedae Glaucae Herba*	Slightly salty, cool-natured	Clearing heat, removing food retention. Mainly treating food retention and fever	Whole herb	*Suaeda glauca* (Bunge) Bunge	The coastal areas of Shandong, Jiangsu, and Zhejiang, China; Korean Peninsula, and Japan.
11	*Suaedae Salsae Herba seu Semen*	Slightly salty, cool-natured	Seed oil: available for patients suffered from hypertension, coronary heart disease, hyperlipidemia.	Whole herb and seeds	*Suaeda salsa* (L.) Pall.	The coastal areas of Hebei, Shandong, Jiangsu, and Zhejiang, China; other areas of Asian.
12	*Acanthi Ilicifolii Radix seu Herba*	Slightly bitter, cool-natured	Clearing heat and detoxifying, eliminating stasis to subdue swelling, relieving pain, dissipating phlegm and eliminating dampness, Relieving cough and asthma. Mainly treating parotic swelling, crewels, splenohepatomegalia, acute and chronic hepatitis, stomachache, lumbar muscle strain, phlegm heat and cough with asthma, jaundice, gonoblennorrhea.	Root or branches and leaves	*Acanthus ilicifolius* L.	The coastal areas of Fujian, Guangdong, Guangxi, and Hainan, China.
13	*Glehniae Radix*	Sweet and slightly bitter in flavor, cool-natured	Nourishing Yin to clear away lung-heat, benefiting stomach and promoting the secretion of saliva, invigorating asthenia and clearing heat, moistening lung to arrest cough. Mainly treating lung-heat and irritating dry cough, over-strained cough and bloody phlegm, consumptive disease and chronic dry cough, pulmonary tuberculosis, chronic bronchitis, lung dryness, consumptive lung disease, deficiency of stomach-Yin, saliva deficiency due to pyreticosis, dry pharynx and thirst, lung cancer.	Root	*Glehnia littoralis* Fr. Schmidt ex Miq.	The coastal areas of Liaoning, Hebei, Shandong, Jiangsu, Zhejiang, Fujian, Guangdong, Hainan, and Taiwan; also planted in Shandong, Fujian, and Inner Mongolia, China; Korean Peninsula, Japan, and Russia.
**Animals**						
14	*Rhopilema*	Salty flavor, mild-natured	Dissipating phlegm and removing stagnation, dispelling wind and eliminating dampness, detumescence by detoxification; mainly treating cough and humid asthma, mass in the abdomen, head-wind and innominate toxic swelling, erysipelas, ecthyma, rheumatic arthritis, and leukorrhagia.	Fimbria	*Rhopilema esculenta* Kishinouye	The sea areas from Liaoning to Fujian, China.
					*Rhopilema hispidum* Vanhoeffen	The sea areas from Shantou, Guangdong to Leizhou Peninsula and Weizhou Island, Guangxi, China.
15	*Acropora*	Acrid flavor, mild-natured	Dispelling wind and arresting itching, detoxifying and removing blood stasis; mainly treating itch of skin, tinea tonsure, sore and carbuncle, postnatal congestion and block, and urolithiasis.	Coral skeleton	*Acropora pulchra* (Brook)	The sea areas of Nansha Islands, Xisha Islands, Taiwan, Hainnan, and Guangxi, China.
					*Acropora* sp.	The sea areas of Xisha Islands, Nansha Islands, Dongsha Islands, Taiwan, Guangdong, and Hainan, China.
					*Acropora ariega* (Dana)	The sea areas of Dongsha Islands, Nansha Islands, Xisha Islands, Taiwan, and Hainan, China.
16	*Tylorrhynchus et Nectoneanthes et Neanthes*	Sweet flavor, warm-natured	Invigorating spleen and stomach, fundoscopic, inducing diuresis to alleviate edema. Mainly treating weakness of the spleen and stomach, dyspepsia, diarrhea, anemia, edema, difficulty in micturition, scabies, dermatophytosis.	Whole body	*Tylorrhynchus heterochaetus* (Quatrefages)	The estuary areas of the Yellow Sea, the East China Sea, and the South China Sea, China; Indonesia, Vietnam, Japan, and the far-eastern Russia.
					*Nectoneanthes oxypoda* (Marenzeller)	The coastal areas of China; Japan, Korean Peninsula, Australia, and New Zealand.
					*Neanthes japonica* (Izuka)	The Bohai Sea, the Yellow Sea, and the East China Sea, China; the coastal areas of Korean Peninsula, and Japan.
17	*Haliotidis Concha*	Salty flavor, cold-natured	Calming the liver and suppressing Yang, clearing heat and calming wind, improving eyesight and clearing nebula, reducing blood pressure, dredging stranguria. Mainly treating headache and dizziness, conjunctival congestion and nephelium, dim-sighted, glaucoma and night blindness, wind-heat in liver and lung, hectic fever due to Yin-deficiency, five kinds of stranguria, hyperacidity, hypertension, apoplexia.	Conch	*Haliotis diversicolor* Reeve	The East China Sea, and the South China Sea, China; the tropical sea areas of the Indian Ocean and the Pacific Ocean.
					*Haliotis discus hannai* Ino	The coastal areas of Liaoning and Shandong Peninsula, China.
					*Haliotis asinina* Linnaeus	The South China Sea, China; The coastal areas of Japan, Philippines, Malaysia, and Australia.
					*Haliotis ovina* Gmelin	The East China Sea, the South China Sea, China.
					*Haliotis laevigata* Donovan	The southwest sea area of Australia.
					*Haliotis ruber* Leach	The coastal areas of Australia; aquiculture in the coastal areas of Guangdong, and Hainan, China.
18	*Haliotidis Musculus*	Sweet and salty in flavor, mild-natured	Nourishing Yin and clearing heat, replenishing vital essence to improve eyesight, nourishing blood and liver, regulating menstruation and lactogenesis, moisturizing dryness and stimulating appetite, benefiting intestines and dredging stranguria. Mainly treating consumptive fever and hectic fever due to Yin-deficiency, cough, glaucoma and cataracta, Irregular menstruation, metrorrhagia and morbid leukorrhea, hypogalactia after delivery, stranguria and turbid discharge, kidney asthenia, frequent urination, dry stool.	Meat	*Haliotis diversicolor* Reeve	The East China Sea, and the South China Sea, China; the tropical sea areas of the Indian Ocean and the Pacific Ocean.
					*Haliotis discus hannai* Ino	The coastal areas of Liaoning and Shandong Peninsula, China.
					*Haliotis asinina* Linnaeus	The South China Sea, China; The coastal areas of Japan, Philippines, Malaysia, and Australia.
					*Haliotis ovina* Gmelin	The East China Sea, the South China Sea, China.
					*Haliotis laevigata* Donovan	The southwest sea area of Australia.
					*Haliotis ruber* Leach	The coastal areas of Australia; aquiculture in the coastal areas of Guangdong, and Hainan, China.
19	*Turbinis Operculum*	Salty flavor, mild-natured	Clearing muggy, eliminating pyrophlegm, Clearing sore-toxin, Clearing hepatic fire, checking diarrhea and dysentery. Mainly treating pain in gastric cavity and abdomen, hematochezia and dysentery, difficulty and pain in micturition, headache, hemorrhoids and fistula, head-sore, ungual gangrence, mange, hypertension.	Operculum	*Turbo cornutus* Solander	The coastal areas from Zhejiang to Hainan, China; from the southern part of Hokkaido to Kyushu, Japan; the southern part of Korean Peninsula.
					*Turbo marmoratus* Linnaeus	The coastal areas of Taiwan and Hainan, the sea areas of Xisha Islands and Nansha Islands, China; Philippines, Amami-Oshima, Japan, Indonesia, the northern part of Australia, Great Barrier Reef, Andaman Islands, and Seychelles Islands.
					*Turbo bruneus* (Röding)	The coastal areas of Guangdong and Hainan, China; Japan, Philippines, Sri Lanka, Fiji Islands, Indonesia, Australia, and the Indian Ocean.
					*Turbo chryostomus* Linnaeus	The coastal areas of Taiwan and Hainan, China; the sea areas of Japan, Philippines, Indonesia, Solomon Islands, Fiji Islands, New Caledonia, Australia, Nicobar Islands, and South Africa.
					*Turbo petholatus* Linnaeus	The sea areas of Xisha Islands Nansha Islands, and Taiwan, China; Philippines, and Amami-Oshima, Japan.
					*Turbo argyrostomus* Linnaeus	The sea areas of Xisha Islands, Nansha Islands, and Taiwan, China; Philippines, and Ban Tak Islands.
*20*	*Rapanae Musculus*	Sweet flavor, cool-natured	Removing heat to brighten vision. Mainly treating liver heat and conjunctival congestion, ophthalmalgia, epigastrium and abdomen thermalgia.	Meat	*Rapana venosa* (Valenciennes)	The coastal areas from Liaoning to Fujian, China.
					*Rapana bezoa*r (Linnaeus)	The coastal areas of Guangdong and Hainan, China.
21	*Rapanae Concha*	Salty flavor, cold-natured	Relieving hyperacidity and analgesia, dissipating phlegm and removing qi stagnation, calming liver wind. Mainly treating gastric and duodenal ulcer, neurasthenia, contracture of hands and feet, chronic osteomyelitis, scrofula.	Conch	*Rapana venosa* (Valenciennes)	The coastal areas from Liaoning to Fujian, China.
					*Rapana bezoa*r (Linnaeus)	The coastal areas of Guangdong and Hainan, China.
22	*Arcae Concha*	Sweet and salty in flavor, mild-natured	Dissolving phlegm and breaking stagnate, dissipation of mass and removing food retention, removing stasis and relieving pain, relieving hyperacidity, Relieving cough, stop dysentery, stop bleeding. Mainly treating intestine obstruction and abdominal mass, phlegmatic mass, chronic cough, crewels, goiter and tumor, epigastralgia, epigastic upset, acid regurgitation, diarrhea, ulcerative gingivitis, bleeding wound, chilblain, burn and scald.	Conch	*Scapharca broughtonii* (Schrenck)	The northern part of the Yellow Sea, China; The sea areas of far-eastern Russia, Japan, and Korean Peninsula.
					*Tegillarca granosa* (Linnaeus)	The coastal areas of China; the sea areas from Indian to the western part of the Pacific Ocean.
					*Scapharca kagoshimensis* (Tokunaga)	The Bohai Sea, the Yellow Sea, the East China Sea, China; the coastal areas of Japan, and Korean Peninsula.
23	*Mytili et Pernae Musculus*	Sweet and salty in flavor, warm-natured	Invigorating the liver and kidney, nourishing Yin and calming wind, nourishing blood and regulating menstruation, boosting essence and marrow, softening hard mass and eliminating stagnation, stop bleeding and diarrhea. Mainly treating consumptive disease and emaciation, dizziness, night sweat, impotence and prospermia, lumbago due to kidney-asthenia, anemia, chronic dysentery, hematemesis, uterine bleeding, morbid leukorrhea, thyroid swelling.	Meat	*Mytilus galloprovincialis* Lamarck	The Yellow Sea, the Bohai Sea, China; sea areas of the Northern Hemisphere, and Oceania.
					*Mytilus coruscus* Gould	The Yellow Sea, the Bohai Sea and the East China Sea, China; the coastal areas of Japan and Korean Peninsula.
					*Perna viridis* (Linnaeus)	The coastal areas from Taiwan Strait to Hainan, China; the Southeast Asia and the Indian Ocean.
					*Trichomya hirsutus* (Lamarck)	The sea areas from Nanji Island in Zhejiang to Hainan, China; Japan, Southeast Asia, India, and Australia.
					*Septifer bilocularis* (Linnaeus)	The South China Sea, China; the sea areas from the southern part of Japan to Australia; the Indian Ocean.
					*Septifer excisus* (Wiegmann)	The sea area from Nanji Island in Zhejiang to Hainan, China; Japan, and Vietnam; the Indian Ocean.
24	*Margarita*	Sweet and salty in flavor, cold-natured	Calming heart and nerves, clearing liver and improving vision, calming wind and arresting convulsion, nourishing the skin, detoxifying and promoting granulation. Mainly treating pavor and palpitation, insomnia and irritability, infantile convulsions and epilepsy, conjunctival congestion and nephelium, aphtha.	Pearl formed by stimulating in mantle of shell	*Pinctada fucata martensii* (Dunker)	The coastal areas of Guangdong, Guangxi, Hainan, and Taiwan, China; the southern part of Japan.
					*Pinctada margaritifera* (Linnaeus)	The sea areas of Guangdong, Guangxi and Taiwan, and Xisha Islands, China; from the Indian Ocean to the western part of the Pacific Ocean.
					*Pinctada maxima* (Jameson)	The sea areas of Taiwan, Hainan, Leizhou Peninsula and Xisha Islands, China; the western part of the Pacific Ocean.
25	*Margaritifera Concha*	Salty flavor, cool-natured	Calming the liver and suppressing Yang, Soothing the nerves and arresting convulsion, Reducing phlegm and removing qi stagnation, calming nausea and preventing vomiting, relieving cough, stop drowsy, stop bleeding, improving eyesight and clearing nebula, removing maculae and nourishing skin. Mainly treating headache and dizziness, conjunctival congestion and tinnitus, palpitation and insomnia, irritability and coma, epilepsy, phlegm and retained fluid, cough and regurgitation.	Prysmatic layer and pearl layer of shell	*Pinctada fucata martensii* (Dunker)	The coastal areas of Guangdong, Guangxi, Hainan, and Taiwan, China; the southern part of Japan.
					*Pinctada margaritifera* (Linnaeus)	The sea areas of Guangdong, Guangxi, Taiwan, and Xisha Islands, China; the Indian Ocean to the western part of the Pacific Ocean.
					*Pinctada maxima* (Jameson)	The sea areas of Taiwan, Hainan, Leizhou Peninsula, and Xisha Islands, China; the western part of the Pacific Ocean.
26	*Ostreae Concha*	Salty and astringent in flavor, slightly cold-natured	Calming the liver and suppressing Yang, mind-tranquilizing, calming wind and stop spasm, clearing heat and removing phlegm, resolving stagnation and removing abdominal mass, softening hard mass and eliminating stagnation, inducing astringency, relieving hyperacidity, quenching thirst, anti-tumor. Mainly treating dizziness and tinnitus, headache, tremor of hands and feet, pavor and insomnia, dysphoria, epilepsy, crewels and goiter, intestine obstruction and abdominal mass, agglomeration in breast, spontaneous perspiration and night sweat, spermatorrhea, frequent urination and, uterine bleeding, morbid leucorrhea, acid regurgitation and stomachache, wasting-thirst, carcinoma.	Conch	*Ostrea gigas* Thunberg	The coastal areas of China; the western part of the Pacific Ocean.
					*Ostrea tahenwhanensis* Crosse	The coastal areas of China; the western part of the Pacific Ocean.
					*Ostrea rivularis* Gould	The estuaries of the coast of China; Japan.
27	*Mactrae et Lutrariae Musculus*	Salty flavor, cold-natured	Nourishing Yin and clearing heat, inducing diuresis to alleviate edema, softening hard mass, eliminating phlegm. Mainly treating wasting-thirst, edema, difficult urination, jaundice, chronic hepatitis, anemia, accumulating phlegm, abdominal mass.	Meat	*Mactra veneriformis* Reeve	The coastal areas of China; Korean Peninsula, and Japan.
					*Lutraria australis* Reeve	The coastal areas of Fujian, Hainan, and Taiwan, China; Vietnam, Philippines, and Australia.
					*Mactra mera* Reeve	The coastal areas of Guangdong, Guangxi, Hainan, China.
28	*Meretricis Concha*	Bitter and salty in flavor, slightly cold-natured	Clearing lung and eliminating phlegm, softening hard mass and eliminating stagnation, inducing diuresis to alleviate edema, relieving hyperacidity and analgesia, restraining sore and eliminating dampness.	Conch	*Meretrix meretrix* (Linnaeus)	The coastal areas from Zhejiang to Hainan, China; Japan, Philippines, Vietnam, Indonesia, India, and Pakistan.
					*Cyclina sinensis* (Gmelin)	The coastal areas of China; Japan, Korean Peninsula, and the western part of the Pacific Ocean.
					*Meretrix lusoria* (Röding)	The coastal areas from Jiangsu to Hainan, China; Japan.
					*Dosinia japonica* (Reeve)	The coastal areas China; the far-eastern Russia, Korean Peninsula, Japan, and Vietnam.
					*Saxidomus purpurata* (Sowerby)	The coastal areas of Liaoning, Hebei, and Shandong, China; Japan, and Korean Peninsula.
29	*Sepiae Endoconcha*	Salty and astringent in flavor, slightly warm-natured	Astringency and hemostasis, arresting spontaneous emission and leukorrhagia, relieving hyperacidity and analgesia, astringing dampness and restraining sore, promoting meridians, relieving cold-dampness, eliminating phlegm, relieving vision nebula. Mainly treating hematemesis, uterine bleeding, hemafecia, bleeding caused by trauma, emission and straguria with turbid discharge, leukorrhea with reddish discharge, hemorrhagic amenorrhea, stomachache and acid regurgitation.	Endoconch	*Sepiella japonica* Sasaki	The Chinese Seas; the Indian Ocean and the western part of the Pacific Ocean.
					*Sepia esculenta* Hoyle	The Chinese Seas; the Philippines Islands.
30	*Octopus*	Sweet and salty in flavor, mild-natured	Nourishing and strengthening body, reinforcing qi and nourishing blood, suppressing dysmenorrhea and promoting lactation, detoxifying and promoting granulation. Mainly treating qi and blood weakness, blood deficiency and blocked menstruation, lactation deficiency after delivery, carbuncle-abscess and swelling-toxicum, chronic sore.	Meat	*Octopus vulgaris* Cuvier	The East China Sea, and the South China Sea, China; wide distribution in all of the oceans.
					*Octopus minor*(Sasaki)	The Chinese Seas; Japan.
					*Octopus fangsiao d’*Orbigny	The Chinese Seas; Japan.
					*Octopus ovulum* (Sasaki)	The East China Sea and the South China Sea, China; the southern sea area of Japan.
31	*Fenneropenaeus et Trachypenaeus*	Sweet and salty in flavor, warm-natured	Boosting constitution and essence, invigorating kidney and rising Yang, nourishing Yin and calming wind, detoxifying, promoting lactation and eruption. Mainly treating impotence due to deficiency of the kidney, and, stirring of wind due to deficiency of Yin, apoplexy, tendon and bone pain, Lactation stoppage, measles.	Meat or whole body	*Fenneropenaeus chinensis* (Osbeck)	The Bohai Sea, the Yellow Sea, and the East China Sea, China; the west coastal area of Korean Peninsula.
					*Fenneropenaeus merguiensis* (De Man)	The coastal areas of Fujian, Guangdong, and Guangxi, China; India, Pakistan, and Burma.
					*Fenneropenaeus penicillatus* (Alcock)	The sea area from Zhoushan Islands in the East China Sea to the South China Sea, China; Pakistan, Buema, and Arab Ocean.
					*Trachypenaeus curvirostris* (Stimpson)	The Bohai Sea, the Yellow Sea, the East China Sea, and the South China Sea.
					*Penaeus monodon* Fabricius	The East China Sea, and the South China Sea, China; Japan, Southeast Asia, and East Africa.
32	*Eriocheir et Gaetice*	Salty flavor, cold-natured	Clearing heat and resolving stagnation, disintegrating blood stasis and promoting menses, subsiding swelling and detoxifying, removing food retention and abortion. Mainly treating jaundice due to damp-heat, stasis and stomachache after delivery, amenorrhea and abdominal pain, tendon and bone damage, carbuncle and furunculosis, scald.	Meat and viscera	*Eriocheir sinensis* H. Milne Edwards	The coastal area of China; the west coastal area of Korean Peninsula.
					*Eriocheir japonica* De Haan	The coastal areas of Fujian, Taiwan, and Guangdong, China; the east coast of Korean Peninsula, and Japan.
					*Gaetice depressus* (De Haan)	The Yellow Sea, the East China Sea, and the South China Sea, China; Korean Peninsula, and Japan.
					*Hemigrapsus penicillatus* (De Haan)	The coastal areas of China; Korean Peninsula, and Japan.
33	*Apostichopus et Stichopus et Thelenota*	Sweet and salty in flavor, mild-natured	Nourishing the kidney and strengthening the essence, strengthening Yang and cure impotence, nourishing blood and promoting the secretion of saliva, regulating menstruation and nourishing the fetus, moisturizing dryness and smoothening intestines, hemostasis, reinforcing consumptive disease. Mainly treating kidney asthenia and damage of essence, impotence, emission, consumptive disease, deficiency of essence and blood, Yin deficiency and fatigue thin, lung asthenia and chronic cough, irritating dry cough, weakness after delivery or disease.	Whole body	*Apostichopus japonicus* (Selenka)	The Yellow Sea, and the Bohai Sea, China; Japan, and Korean Peninsula.
					*Stichopus variegatus* Semper	The sea areas of Guangdong, Guangxi, Hainan, Taiwan, and Xisha islands, China.
					*Stichopus horrens* Selenka	The sea areas of Hainan, Taiwan, and Xisha Islands, China; Madagascar, New Caledonia, Philippines, and Indonesia.
					*Stichopus chloronotus* Brandt	The sea areas of Hainan, Xisha Islands, Zhongsha Islands, and Nansha Islands, China; the Indian Ocean to the western part of the Pacific Ocean.
					*Thelenota ananas* (Jaeger)	The sea areas of Taiwan, Xisha Islands, and Nansha Islands, China; East Africa, and Madagascar.
34	*Craspidaster*	Salty flavor, mild-natured	Clearing heat and detoxifying, resolving hard lump, harmonizing stomach and relieving pain. Mainly treating thyroid swelling, scrofula, crewels, stomachache and acid regurgitation, diarrhea, Otitis media.	Whole body	*Craspidaster hesperus* (Müller *et* Troschel)	The coastal areas of Zhejiang, Fujian, and Guangdong, China; the southern part of Japan, Singapore, and Philippines.
					*Stellaster equestris* (Retzius)	The South China Sea and the East China Sea, China; the Indian Ocean to the western part of the Pacific Ocean.
					*Anthenea pentagonula* (Lamarck)	The coastal areas of Guangdong, and Fujian, China.
					*Rosaster symbolicus* (Sladen)	The eastern sea area of Hainan Island, China; Arafura Sea, Ban Tak Sea, and Philippines Sea.
35	*Asterina*	Salty flavor, warm-natured	Expelling rheumatism, relieving pain, invigorating kidney, strengthening Yang, relieving hyperacidity. Mainly treating rheumatism pain in waist and lower extremities, stomachache and acid regurgitation, impotence.	Whole body	*Asterina pectinifera* (Müller *et* Troschel)	The Bohai Sea, and the Yellow Sea, China; the far-eastern sea area of Russia, Japan, and Korean Peninsula.
					*Asterina limboonkengi* G. A. Smith	The coastal areas of Guangdong, and Fujian, China.
					*Asterina batheri* Goto	The offshore area of Yantai, China; the coastal area from Honshu to Kyushu, Japan.
36	*Asterias*	Salty flavor, mild-natured	Harmonizing stomach and relieving pain, clearing heat and detoxifying, Calming the liver and relieving convulsion, softening hard mass and eliminating stagnation. Mainly treating stomachache and acid regurgitation, gastric ulcer and duodenal ulcer, diarrhea, epilepsy, crewels, otitis media.	Whole body	*Asterias rollestoni* Bell	The Yellow Sea, and the Bohai Sea, China; the far-eastern sea area of Russia, and the coastal area of Japan.
					*Asterias amurensis* Lütken	The Yellow Sea, and the Bohai Sea, China; the coastal areas of Asia in the northern Pacific Ocean.
					*Aphelasterias changfengYingi* Baranova *et* Wu	The Bohai Sea, and the northern part of Yellow Sea, China.
					*Asterias argonauta* Djakonov	The Bohai Sea, China; the southern part of Japan Sea, and Korean Peninsula.
					*Asterias versicolor* Sladen	The Bohai Sea, and the northern part of the Yellow Sea, China; Japan Sea and the southern seacoast of Japan.
37	*Anthocidaris et Hemicentrotus et Strongylocentrotus Concha*	Salty flavor, mild-natured	Softening hard mass and eliminating phlegm, Removing stasis and swelling, relieving hyperacidity and analgesia, clearing heat and detoxifying. Mainly treating tuberculosis of cervical lymph nodes, crewels and subcutaneous nodule, goiter and tumor, asthma, swelling pain in sternal ribs, stomachache, paronychia.	Calcareous bone shell	*Hemicentrotus pulcherrimus* (A. Agassiz)	The Bohai Sea, the Yellow Sea, and the East Sea, China; Japan.
					*Strongylocentrotus nudus* (A. Agassiz)	The coastal areas of Liaoning Peninsula and the northern part of Shandong Peninsula, China; Japan, Russia.
					*Anthocidaris crassispina* (A. Agassiz)	The coastal areas of Zhejiang, Fujian, Guangdong, Taiwan and Hainan, China; the southern part of Japan Sea.
					*Temnopleurus toreumaticus* (Leske)	The Chinese Seas; the Indian Ocean to the western part of the Pacific Ocean.
					*Temnopleurus hardwickii* (Gray)	The northern coastal areas of Fujian, China; Japan, and Korean Peninsula.
38	*Syngnathus*	Sweet and salty in flavor, warm-natured	Invigorating the kidney and strengthening Yang, Removing stasis and swelling, relaxing and activating the tendons, relieving pain, hemostasis, expediting child delivery, antifatigue, anti-aging, anti-tumor. Mainly treating impotence, infertility, emission, infertility due to cold uterus, dystocia, kidney asthenia and asthma, rheumatism and paralysis pain, intestine obstruction and accumulation, crewels and goiter, traumatic injury, scrofula, carbuncle and furunculosis.	Whole body or whole body removed skin and viscera	*Syngnathus acus* Linnaeus	The coastal areas of Shandong, China; the southern part of Korean Peninsula, and Japan.
					*Syngnathoides biaculeatus* (Bloch)	The East China Sea, and the South China Sea, China; Japan, Philippines, and Indonesia.
					*Solegnathus hardwickii* (Gray)	The East China Sea, and the South China Sea, China; the coastal areas of Japan, India, New Zealand and Africa.
39	*Hippocampus*	Sweet and salty in flavor, warm-natured	Invigorating the kidney and strengthening Yang, boosting essence, relieving cough and asthma, promoting blood circulation to remove meridian obstruction, removing stasis and subsiding swelling, induced abortion. Mainly treating deficiency of the kidney asthenia and damage of essence, impotence and infertility, infertility due to cold uterus, dyspnea due to deficiency, chronic asthma, enuresis, deficient dysphoria and insomnia, intestine obstruction and accumulation, abdominal mass, stasis and stomachache, traumatic injury, bleeding wound, carbuncle and furuncle, swelling-toxicum, dystocia, scrofula and thyroid swelling.	Whole body removed viscera	*Hippocampus trimaculatus* Leach	The East China Sea, and the South China Sea, China.
					*Hippocampus kelloggi* Jordan *et* Snyder	The East China Sea, and the South China Sea, China; Japan, and Vietnam.
					*Hippocampus histrix* Kaup	The East China Sea and the South China Sea, China; Japan, Vietnam, Indonesia, and Philippines.
					*Hippocampus kuda* Bleeker	The East China Sea, and the South China Sea, China; Japan, Philippines, Malaysia, and Indonesia.
					*Hippocampus japonicus* Kaup	The coastal areas of China, mainly in Liaoning, Hebei, Shandong, and Zhejiang, China; Korean Peninsula, and Japan.
40	*Lateolabrax et Cephalopholis et Plectropomus*	Sweet flavor, mild-natured	Strengthening the spleen and stomach, benefiting liver and kidney, invigorating qi and soothing fetus, dispelling cold and checking diarrhea, relieving cough and reducing sputum, inducing diuresis to alleviate edema. Mainly treating splenasthenic diarrhea, dyspepsia, infantile malnutrition, pertussis, edema, migratory arthralgia, weakness of muscles and bones, fetal irritability, hypogalactia after delivery, chronic sore and ulcer.	Meat or whole body	*Lateolabrax maculatus* (McClelland)	The coastal area of China, mainly in estuary areas.
					*Cephalopholis argus* Bloch *et* Schneider	The islands of the South China Sea, and the sea area of Taiwan, China.
					*Plectropomus areolatus* Rüppell	The islands of the South China Sea, and the sea area of Taiwan, China.
41	*Larimichthys*	Sweet flavor, mild-natured	Appetizing and promoting digestion, replenishing qi to invigorate the spleen, nourishing liver and kidney, improving eyesight and soothing the nerves, detoxifying and relieve dysentery. Mainly treating weakness after disease or delivery, hypogalactia, lumbago due to kidney-asthenia, edema, dim-sighted, headache, stomachache, abdominal distension due to dyspepsia, inappetence, dyspepsia, dysentery.	Meat	*Larimichthys crocea* (Richardson)	The southern part of Yellow Sea, the East China Sea, and the South China Sea (until Qiongzhou Strait), China; the southwest sea area of Korean Peninsula.
					*Larimichthys polyactis* (Bleeker)	The Bohai Sea, the Yellow Sea, and the East China Sea; the southwest sea area of Korean Peninsula.
42	*Larimichthys Auris Lithos*	Sweet and salty in flavor, cold-natured	Inducing diuresis for treating stranguria, clearing heat and detoxifying. Mainly treating urolithiasis, dribbling urination, cholelithiasis, rhinitis, suppurative otitis media; food and drug poisoning.	Otolith	*Larimichthys crocea* (Richardson)	The southern part of the Yellow Sea, the East China Sea, the South China Sea (until Qiongzhou Strait), China; the southwest sea area of Korean Peninsula.
					*Larimichthys polyactis* (Bleeker)	The Bohai Sea, the Yellow Sea, and the East China Sea; the southwest sea area of Korea.
43	*Scomber*	Sweet flavor, mild-natured	Nourishing and strengthening body, invigorating lung and reinforcing kidney, strengthening spleen and appetizing. Mainly treating weakness of spleen and stomach, dyspepsia, vomiting and diarrhea, tuberculosis, kidney asthenia, neurasthenia.	Meat or whole body	*Scomber japonicus* Houttuyn	The coastal area of China; the western part of the Pacific Ocean.
					*Scomber australasicus* Cuvier	The southern part of the East China Sea, and Taiwan Strait, China; Korean Peninsula, and Japan.
44	*Pampus et Psenopsis*	Sweet flavor, warm-natured	Reinforcing qi and nourishing blood, warming kidney and invigorating Yang, relaxing tendon and benefiting bone, Mainly treating weakness of spleen and stomach, dyspepsia, anemia, weakness after illness, inability of legs and feet, soreness in bones and muscles, numbness of limbs.	Meat or whole body	*Pampus argenteus* (Euphrasen)	The coastal area of China; Korean Peninsula, and Indonesia.
					*Pampus chinensis* (Euphrasen)	The southern part of the East China Sea, and the South China Sea, China; Japan.
					*Pampus cinereus* (Bloch)	The southern part of the East China Sea, and the South China Sea, China; Japan.
					*Psenopsis anomala* (Temminck *et* Schlegel)	The southern part of the East China Sea, and the South China Sea, China; Japan, and Korean Peninsula.
45	*Pegasus et Eurypegasus*	Sweet flavor, mild-natured	Eliminating phlegm and relieving coughing, removing and goiter stasis, invigorating kidney and strengthening Yang, strengthening spleen and checking diarrhea. Mainly treating infantile expectoration, trachitis, measles, diarrhea after measles, goiter and subcutaneous nodule, thyroid tumor, impotence due to deficiency of the kidney.	Meat or whole body	*Pegasus laternarius* Cuvier	The East China Sea, and the South China Sea, China; Japan.
					*Pegasus volitans* Linnaeus	The East China Sea, the South China Sea, and the coast of Taiwan, China.
					*Eurypegasus draconis* (Linnaeus)	The South China Sea, and the coast of Taiwan, China.
46	*Eretmochelys*	Sweet and salty in flavor, cold-natured	Calming liver and arresting convulsion, clearing heat and detoxifying, dispelling heat and improving eyesight. Mainly treating aphasia from apoplexy, fever and hyperpyrexia, coma and delirium, convulsion, infantile convulsive epilepsy, dizziness, upset and insomnia, carbuncle and furunculosis, hypertension.	Carapace	*Eretmochelys imbricata* (Linnaeus)	The coastal areas of Shandong, Jiangsu, Zhejiang, Fujian, Guangdong, Guangxi, Taiwan, Hainan, and islands in the South China Sea, China; tropical and subtropical sea areas.
47	*Hydrophis et Laticauda et Lapemis*	Sweet and salty in flavor, mild-natured	Dispelling wind and eliminating dampness, promoting blood circulation to remove meridian obstruction, nourishing qi and blood, strengthening tendon and bone, relieving swelling and pain, detoxifying and stopping dysentery, relieving cough and asthma, eliminating dampness and alleviating itching, nourishing skin. Mainly treating rheumatism and paralysis pain, numbness of hands and feet, soreness in waist and knee, hemiplegia, rheumatoid arthritis, rheumatic arthritis, tetanus, convulsive epilepsy, scrofula, dysentery, bronchitis, leprosy and malignant sore, furuncle, skin itch, chronic eczema, mange, sore of mixed hemorrhoids.	Dried body with viscera removed	*Hydrophis cyanocinuctus* Daudin	The China Sea; the sea areas from the Persian Gulf, passing through Indian Peninsula to Japan and Australia.
					*Hydrophis caerulescens* (Shaw)	The coastal areas of Shandong, Guangdong, and Taiwan, China; the sea areas from the Indian Ocean, passing through the South China Sea to Indonesia and the northern sea area of Australia.
					*Hydrophis fasciatus* (Schneider)	The coastal area of Zhejiang, Fujian, and Taiwan, China; Japan Islands.
					*Hydrophis melanocephalus* Gray	The coastal areas of Zhejiang, Fujian, and Taiwan, China; Japan Islands.
					*Hydrophis ornatus* (Gray)	The coastal areas of Shandong, Guangdong, Guangxi, Hainan, and Taiwan, China; the sea areas from the Persian Gulf, passing through Indian Peninsula to Australia.
					*Hydrophis gracilis* (Shaw)	The coastal areas of Fujian, Guangdong, Guangxi, and Hainan, China; the sea areas from the Persian Gulf, passing through Indian Peninsula, Australia To Papua New Guinea.
					*Laticauda semifasciata* (Reinwardt)	The coastal areas of Liaoning, Fujian, and Taiwan, China.
					*Lapemis curtus* (Shaw)	The coastal areas of Shandong, Fujian, Taiwan, Hong Kong, Hainan, and Guangxi, China.
**Minerals**						
48	*Costazia et Celleporina*	Salty, cold-natured	Clearing lung and eliminating phlegm, softening hard mass and eliminating stagnation. Mainly treating phlegm-heat and cough, goiter and tumor, swelling sore.	Bone	*Costazia aculeate* Canu *et* Bassler	The southern coastal area of China.
					*Celleporina costazii* (Audouin)	The coastal areas of Shandong Peninsula, Jiangsu, Zhejiang, Fujian, Guangdong, Hainan, Xisha Islands, Zhongsha Islands, and Nansha Islands.
49	*Galaxea et Balanop*	Sweet, warm-natured	Warming lung and depressing qi, strengthening Yang and promoting lactation. Mainly treating lung cold, cough and asthma, impotence, obstructed breast pulse.	Calcareous bone	*Galaxea aspera* Quelch	The coastal areas of Guangxi, Guangdong, and Hainan, China.
					*Galaxea fascicularis* (Linnaeus)	The coastal areas of Guangxi, Guangdong, Hainan, Taiwan, Dongsha Islands, Xisha Islands, and Nansha islands, China.
					*Balanophgllia* sp.	The coastal areas of Guangdong, and Guangxi, China.
50	*Cyrtiospirif*	Sweet and salty in flavor, cold-natured	Eliminating dampness-heat, inducing urination, eliminating nebula. Mainly treating gonorrhea, difficult urination, morbid leukorrhea, hemuresis, dim eyesight due to nephelium.	Fossil	*Cyrtiospirifer sinensis* (Graban)	Mainly produced in Hunan, China.

## Supplementary Files

Supplementary File 1
