# Supplementary material for: Chinese Marine Materia Medica Resources: Status and Potential
Source: Mar Drugs. 2016 Mar 3;14(3):46. doi: 10.3390/md14030046 (PMC4820300; doi:10.3390/md14030046)
Supplement: Supplementary File 1 [file marinedrugs-14-00046-s001.pdf]

Supplementary Materials: Chinese Marine *Materia Medica* Resources: Status and Potential

Xiu-Mei Fu, Meng-Qi Zhang, Chang-Lun Shao, Guo-Qiang Li, Hong Bai, Gui-Lin Dai, Qian-Wen Chen, Wei Kong, Xian-Jun Fu and Chang-Yun Wang

The questionnaire of traditional Chinese medicine commodity of "Excavation and Rectification of Marine Traditional Chinese Medicinal Bioresources"

Survey site: Bozhou TCM Market in Anhui Survey time: 6 May 2013

<table>
<tr><th colspan="2">Drug name</th><th>Quality grade</th><th>Storage condition</th><th>Storage time</th><th>Processing method</th></tr>
<tr><td colspan="2">1-3-1 Syngnathus</td><td>Unkown</td><td>Air drying</td><td>Unkown</td><td>Drying in the sun</td></tr>
<tr><th colspan="2">Original organism</th><th>Resource status</th><th>Production area</th><th>Output</th><th>Sales volume</th></tr>
<tr><td colspan="2">Solegnathus hardwickii (Gray)</td><td>□ Cultured
■ Wild</td><td>Hainan</td><td>Unkown</td><td>500–600 kg/Year</td></tr>
<tr><th colspan="2">Medicament portion</th><td colspan="4">Whole body</td></tr>
<tr><th colspan="2">Main function</th><td colspan="4">Invigorating the kidney and strengthening Yang</td></tr>
<tr><th colspan="2">Price（Yuan/kg）</th><td>36000</td><th colspan="2">Price tendency</th><td>□ Rise ■ Stable □ Fall</td></tr>
<tr><th colspan="2">Trading periodicity</th><td colspan="4">■ Slack season □ Busy season</td></tr>
<tr><th colspan="2">Retailer</th><td>Chaofeng pharmacy</td><th>Retailer contact information</th><td colspan="2">13965783596</td></tr>
<tr><th rowspan="2">Usage</th><th>Chinese patent medicine</th><th>Industrial raw materials</th><th>Foodstuff</th><th>Food processing</th><th>Medicinal materials store</th></tr>
<tr><td>√</td><td></td><td></td><td></td><td>√</td></tr>
<tr><th>Remark</th><td colspan="5">The sales volume in summer is lower than autumn.</td></tr>
</table>

Recorder: Mengqi Zhang Verifier: Yunfei Wu Person in charge: Changyun Wang
